# Analytical approaches for the evaluation of data deficient simulated leachable compounds in ENDS products: a case study

**DOI:** 10.3389/fchem.2023.1212744

**Published:** 2023-08-04

**Authors:** Cameron Smith, Matthew Lyndon, Lena Jeong, Danielle Lehman, J. Brian Jameson, Harish Chevva, Felix Ayala-Fierro, David Cook, Karen Carter, Michael Oldham, I. Gene Gillman

**Affiliations:** Juul Labs Inc., Washington, DC, United States

**Keywords:** ENDS, e-cigarette, electronic cigarette, nicotine, leachables, risk assessment, aerosol

## Abstract

Leachable investigations are routinely undertaken across a range of sectors (e.g., pharmaceuticals, medical devices, etc.) to determine whether chemicals from a container closure system transfer into a product under normal conditions of use. For Electronic Nicotine Delivery Systems (ENDS) the container closure system includes all materials in contact with the e-liquid that is aerosolized and subsequently inhaled by the user. Currently, there is no guidance for conducting leachable studies for ENDS products, however, there are relevant guidance documents for orally inhaled drug products that can be applied to an ENDS container closure system. We present a case study of the analytical investigation of two leachable compounds identified in simulated leachable studies using aged JUULpods filled with unflavored e-liquid (PG/VG/nicotine/benzoic acid). Both compounds had limited toxicological information and were considered data deficient. A qualitative analysis of the aerosol collected from aged commercial JUULpods (Virginia Tobacco and Menthol), using a similar analytical method (LC-MS/MS) used in the simulated leachable studies, showed no trace or detectable levels of either leachable compound. Therefore, this qualitative analysis did not provide semi-quantitative values for the data-deficient leachable compounds necessary to support toxicological risk assessment. Further, no commercial authentic standards or reasonable synthetic route were available due to the molecular size and structural complexity of the compounds. Instead, method limits were established using an alternative approach to standard ICH guidelines. The experimentally determined method limit of quantitation, using spiked samples of simulated leachable e-liquid, provided conservative semi-quantitative values for each data deficient leachable compound in the aerosol that enabled a transfer efficiency from e-liquid to aerosol to be estimated. The transfer efficiency of each leachable compound was experimentally determined to be less than 3% based on the limit of quantitation, which then could be used to define a relevant exposure limit for the toxicological risk assessment. This work details a novel analytical approach for determining the transfer efficiency of data deficient leachable compounds from ENDS container closure systems into the ENDS aerosol to support toxicological health risk assessments.

## 1 Introduction

In 2019, the FDA issued guidance for the premarket tobacco product application for Electronic Nicotine Delivery Systems (ENDS) which recommends that manufacturers not only measure specific, or targeted, harmful and potentially harmful constituents (HPHCs), but also recommends manufacturers provide a complete list of uniquely identified, non-targeted, constituents contained within or delivered by the product (USFDA, 2019). ENDS may contain unique compounds that arise from the primary container closure system, i.e., cartridge components, mouthpiece, heating element, etc., that may leach into the e-liquid, potentially resulting in consumer exposure (Wei et al., 2020). Although no formal guidance is available with respect to “uniquely identified” compounds, FDA published a memorandum (USFDA, 2020a) for the evaluation of leachable compounds in tobacco products that recommends ENDS manufacturers determine whether users are exposed, i.e., through aerosol, to these types of compounds by following the published literature (Ball et al., 2007; Norwood et al., 2008), Center for Drug Evaluation and Research (CDER) (USFDA, 2002), International Council for Harmonization of Technical Requirements for Pharmaceuticals for Human Use (ICH) best practices outlined for orally inhaled and nasal drug products (OINDP) (ICH, 2019), as well as United States Pharmacopeia (USP) guidance for study conditions and analytical protocols (USP, 2018; USP, 2020). In general, study conditions to identify compounds that leach from the primary container closure system are straightforward and similar to the aforementioned guidance documents. Extractable studies are designed to identify volatile, semi-volatile, non-volatile inorganic and organic compounds that may be released from individual components (i.e., cartridge components, mouthpiece, heating element, etc.) into the e-liquid under the “worst-case scenario.” Leachable studies assess migration of compounds into the e-liquid under normal conditions that are more relevant to product storage. However, unlike OINDPs that often operate by passive actuation or mechanical nebulization to deliver the active pharmaceutical ingredient without active heating (Donovan et al., 2012; Stein et al., 2014), e-liquid in ENDS products undergoes heating and vaporization to generate a nicotine containing aerosol. Therefore, when studying leachable compounds migrating from the primary container closure system in ENDS, it is important to consider changes that may occur upon heating (e.g., thermal degradation or non-volatility).

Ideally, non-targeted analysis (GC-MS/MS and LC-MS/MS) of e-liquid contained within the ENDS container closure system provides a list of confident, or confirmed, identifications of compounds based on library spectral matches that can be traced back to the source material within the container closure system. The semi-quantitative estimated concentration for each identified leachable compound, above a predetermined analytical evaluation threshold (AET), are subject to toxicological health risk assessment. When relevant inhalation data is available, and quantitative analysis of leachable compounds in the aerosol is necessary for completion of the risk assessment, targeted analytical methods can be performed for each leachable compound in the aerosol using validated methods and authentic standards according to ICH guidelines (ICH, 2005). However, given the variety of unpredictable leachable compounds, the non-targeted analytical methods typically provide tentative leachable identifications in the e-liquid based on mass, molecular formula, and expert mass spectral interpretation. In these cases, toxicologists may employ (Q)SAR assessments (e.g., Derek Nexus, Sarah Nexus, Leadscope Model Applier, etc.), expert judgment, and read-across approaches per FDA guidance and published literature to evaluate the proposed identifications (Broschard et al., 2016). Frequently, identified leachable compounds will not have commercially available standards, nor practical synthetic routes (Sica et al., 2019), making quantitation in the aerosol impractical and often impossible. These difficulties in leachable identification and assessment are common in non-targeted leachable screening and not unique to ENDS products (Jenke, 2020). When reliable quantitative aerosol data is not available, toxicologists conservatively estimate exposure assuming 100% of the identified leachable compound(s) measured in the e-liquid transfers to the aerosol.

Herein, we present a case study of the analytical investigation of two data deficient leachable compounds reported in simulated leachable studies from JUULpods filled with unflavored e-liquid containing PG, VG, nicotine, and benzoic acid. Analytical standards for both compounds were not commercially available, and synthetic routes were impractical due to molecular size and complexity. Therefore, a novel approach was needed to determine whether simulated leachable compounds transferred from e-liquid to aerosol. In addition to transfer efficiency estimations, limits of quantitation (LOQ) for the leachable compounds were experimentally determined. The reported methodology provides an alternative approach for the investigation of leachable compounds, that cannot be assessed by other methods, to support toxicological risk assessment.

## 2 Experimental methods

### 2.1 Case study background: analytical and toxicological summary of simulated leachable studies

Simulated leachable studies were conducted at WuXi AppTec., Inc. (St. Paul, Mn) on JUULpods filled with 5.0% nicotine unflavored e-liquid (propylene glycol, glycerin, nicotine, and benzoic acid) stored at two accelerated conditions (30°C/65% RH and 40°C/70% RH) for 22 weeks simulating 9-month and 18-month aging, respectively (ASTM, 2002). The unflavored e-liquid utilized the same base ingredients as commercial JUULpod formulations, however, unflavored JUULpods are not commercially available. The samples were analyzed by gas chromatography-mass spectrometry (GC-MS) and liquid chromatography-mass spectrometry (LC-MS) in both positive and negative electrospray ionization (ESI) mode to cover a wide range of volatile, semi-volatile and non-volatile organic compounds. For organic compounds, an AET of 0.75 μg/pod (1 pod = 1 device) was used as the reporting threshold calculated based on concepts outlined in PQRI guidelines, however, the threshold of toxicological concern (TTC) value of 1.5 μg/day for mutagenic impurities was used per relevant guidance and standards for medical devices as opposed to the safety concern threshold (SCT) value of 0.15 μg/day more applicable to OINDPS (ISO, 2002; ICH, 2017; ISO, 2019; ISO, 2020). For conversion from the dose-based threshold (DBT, i.e., TTC) to a concentration-based threshold (AET), 1.5 μg/day was divided by a hypothetical worst-case daily consumer exposure of two JUULpods/day. The use of two JUULpods/day was used as a worst case and was consistent with the subsequently calculated median (0.6 pods/day) and 95th percentile (1.3 pods/day) of 12-month JUUL use from clinical study data (12 months after the purchase of a JUUL Starter Kit—and presumably after 12 months of exclusive JUUL use). A toxicological health risk assessment was performed on each compound identified above the AET in the simulated leachable studies according to methodologies and principles outlined by regulatory agencies, as well as national and international standards for container closure systems (ISO, 2002; ICH, 2016; ICH, 2017; USP, 2018; ICH, 2019; USFDA, 2020b; USFDA, 2020c; USP, 2020). All identified leachable compounds estimated concentrations in the e-liquid yielded a margin of exposure (MOE) greater than one utilizing an exposure assumption of 100% transfer from e-liquid to aerosol, with the exception of two data deficient leachable compounds (Table 1). Both compounds were undetected in GC-MS and LC-MS ESI positive mode analyses preventing orthogonal confirmation, and therefore, the limited compound information, proposed structures, and tentative identifications were based on mass spectral interpretation of the LC-MS ESI negative mode analysis.

**TABLE 1:** Information on data deficient leachable compounds.

Name	Compound 1 [RT 1.71 min]	Compound 2 [RT 2.44 min]
CAS #	Not Given	Not Given
Molecular Formula	C_16_H_20_N_2_O_5_	C_18_H_24_N_2_O_4_
Molecular Weight	320.1360	446.1686
Estimated Concentration at 30°C/65%RH for 22 weeks (Simulated 9-month aging)	1.1 ± 0.1 µg/device	2.0 ± 0.1 µg/device
Estimated Concentration at 40°C/75% RH for 22 weeks (Simulated 18-month aging)	8.5 ± 0.7 µg/device	6.2 ± 0.4 µg/device
Structural Characteristics	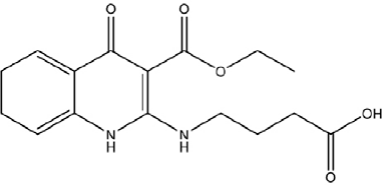	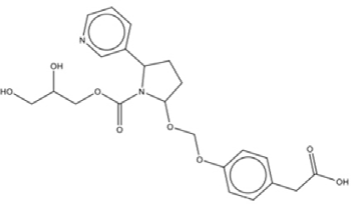
Reported Tentative Compound Identification	1,8,9-Trihydro-2-(3-carboxypropylamine-N-yl)-3-ethylcarboxylate-4-quinolone	Nornicotine, N-carboxyglycerol-5′- [methoxy-1-(ρ-hydroxybenzene-*O*4-yl-acetic acid)]
Abbreviation for Narrative	TCEQ	NNMA

### 2.2 Study design—analytical approach for the investigation of TCEQ and NNMA

**FIGURE 1:**
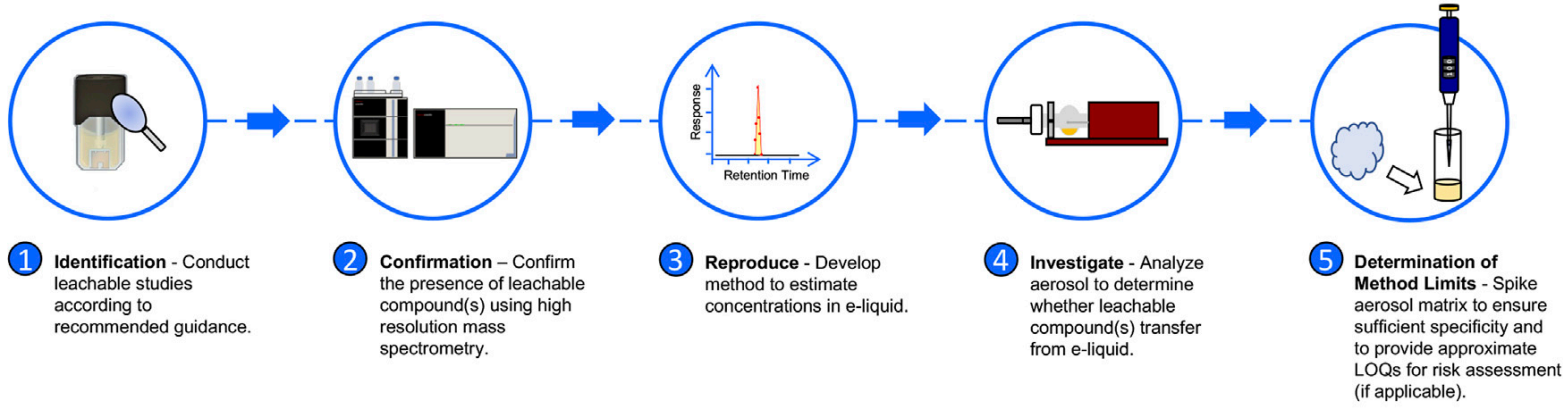
Analytical approach for evaluation of leachable compounds in ENDS products.

Figure 1 depicts the analytical approach to determining whether leachable compounds transfer from e-liquid to aerosol, utilizing LC-HR-MS/MS and LC-MS/MS. Brief details regarding the analytical methods used for the simulated leachable studies conducted by WuXi AppTec., Inc. are provided in the Supplementary Material for reference. All studies, with the exception of the simulated leachable studies discussed in the aforementioned Section 2.1, were performed at the Juul Labs Regulatory Chemistry Laboratory (JLRCL, Durham, NC), an ISO 17025 accredited laboratory. All JUULpods used for analytical investigations at JLRCL, including 5.0% nicotine unflavored e-liquid, commercial Virginia Tobacco JUULpods (5.0% and 3.0% nicotine), and commercial Menthol JUULpods (5.0% and 3.0% nicotine), were stored at ambient conditions (25°C/60% RH) at Precision Stability Storage (Wilson, NC) for a minimum of 2 years. Supplementary Table S1 provides additional information regarding the age of each sample analyzed in the analytical investigation.

### 2.3 e-liquid sample preparation and analysis using LC-HR-MS/MS and LC-MS/MS negative mode ESI

To confirm the presence of compounds TCEQ and NNMA (#2 in Figure 1), 50 mg of unflavored e-liquid was removed from JUULpods aged for 28 months at ambient conditions (25°C/60% RH). The e-liquid was diluted in 1:1 water:methanol and mixed thoroughly with a vortex mixer. The diluted sample was injected into a ThermoFisher Scientific LC Orbitrap ID-X LC-HR-MS/MS (Waltham, MA) equipped with the analytical column, mobile phases, elution gradient, ionization type and mode, as shown in Table 2. Note, mass spectral analysis was consistent with the high-resolution molecular mass and fragmentation patterns with proposed structures and compound rationalizations in Table 1. Upon confirming detectable levels of TCEQ and NNMA in the unflavored e-liquid from aged JUULpods, the method was transferred and optimized (#3 in Figure 1) on an Agilent Technologies Ultivo Triple Quadrupole MS (Santa Clara, CA). The e-liquid sample solution was prepared (*n* = 2) by mixing 50 mg of unflavored e-liquid extracted from aged JUULpods with 1 mL of ultrapure water containing internal standard (ISTD, 40 ppb methylparaben). Instrument parameters for LC-MS/MS analysis used are shown in Table 2.

**TABLE 2:** LC-HR-MS/MS and LC-MS/MS analytical method parameters for analysis of TCEQ and NNMA.

Parameter	JLRCL LC-HR-MS/MS			JLRCL LC-MS/MS		
Instrument	ThermoFisher Scientific Orbitrap ID-X			Agilent Technologies Ultivo TQ		
Ionization	ESI(−)			ESI(−)		
Mobile Phase A	water + 0.1% acetic acid			water + 0.1% acetic acid		
Mobile Phase B	methanol + 0.1% acetic acid			methanol + 0.1% acetic acid		
Flow Rate	0.5 mL/min			0.5 mL/min		
Gradient	Time (mins.)	%A	%B	Time (mins.)	%A	%B
	0.00	95.0	5.0	0.00	95.00	5.00
	0.75	95.0	5.0	0.75	95.00	5.00
	3.00	5.0	95.0	3.00	5.00	95.00
	19.00	5.0	95.0	10.00	5.00	95.00
	19.10	0.0	100.0	10.01	95.00	5.00
	28.00	0.0	100.0	13.00	95.00	5.00
	28.20	95.0	5.0	NA	NA	NA
	30.0	95.0	5.0	NA	NA	NA
Injection Volume	5 µL			1 µL		
Analytical Column	Agilent Technologies Zorbax SB-C18 2.1 × 100mm, 1.8 µm			Agilent Technologies Zorbax SB-C18 2.1 × 100mm, 1.8 µm		
Internal Standard	NA			methylparaben		

### 2.4 Aerosol collection and analysis using LC-MS/MS negative ion mode ESI

**FIGURE 2:**
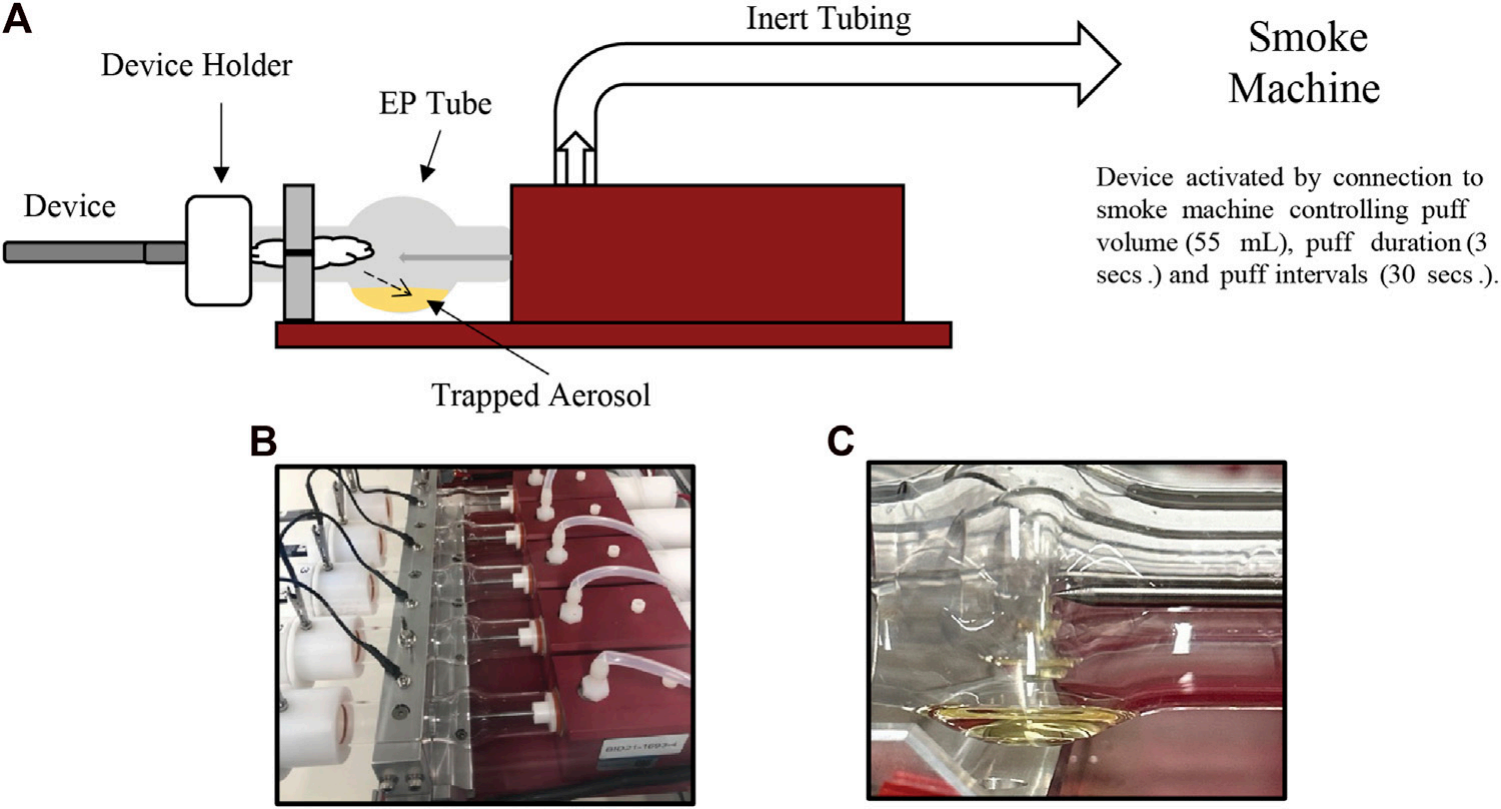
Electrostatic precipitator (EP) system depicting the collection of aerosol from ENDS product **(A)**. The photographs shown are representative graphics of the EP system before **(B)** and after **(C)** collection of aerosol from JUULpods.

Aerosol collections were performed using a Cerulean SM450e puffing machine connected to a Halder Process Solutions HPS-EP5 electrostatic precipitator (EP) system (Halder Process Solutions, Moseley, VA, United States), Figure 2. Aerosol was collected by puffing according to the ISO 20768:2018 standard puffing regime (55 mL, 3 s, 30 s) (ISO, 2018). Each device (fully charged battery) and pod were weighed before and after aerosol collection. Initial collections were performed by collecting 250 puffs. The resulting device mass loss (DML) was calculated by subtracting the post-collection weight from the pre-collection weight. If DML was less than 500 mg, subsequent 50 puff collections were performed until DML was ≥500 mg. After 500 mg of collected aerosol was achieved, individual EP tubes were drained into separate 100 mL DigiTUBEs. The EP tube was rinsed with 5.0 mL of ultrapure water containing ISTD and mixed well after rinsing was complete. The resulting aerosol sample was analyzed using the Agilent Technologies Ultivo Triple Quadrupole LC-MS/MS method as shown in Table 2 (#4 in Figure 1).

### 2.5 Calculation for estimated concentration of TCEQ and NNMA

Estimated TCEQ and NNMA concentrations were calculated using the integrated area of chromatographic peaks from LC-MS/MS analysis according to Eq. 1.

Equation 1: Calculation used for Analytical Reporting of TCEQ and NNMA

$$\frac{\text{Analyte}\;\left(\text{TCEQ}\;\text{or}\;\text{NNMA}\right)\;\text{Peak}\;\text{Area}}{\text{ISTD}\;\text{Peak}\;\text{Area}}\times \text{ISTD Conc}.\left(0.04\frac{\mu g}{mL}\right)\times \frac{\text{Sample}\;\text{Volume}\;\left(\text{mL}\right)}{e-Liquid\;Aliquit\;Wt.\left(g\right)}\times \text{Fill Wt}.\text{in JUULpod}\left(0.780\frac{gram}{Device}\right)=Estimated\;Concentration\;of\;TCEQ\;or\;NNMA\;\left(\frac{\mu g}{\text{Device}}\right)$$ (1)

### 2.6 Determination of transfer efficiency and method limits

Using an aged unflavored JUULpod e-liquid, an investigation of transfer efficiency (#4 in Figure 1) for TCEQ and NNMA was performed according to Eq. 2.

Equation 2: Calculation of Transfer Efficiency for TCEQ and NNMA

$$\frac{\text{Esitmated}\;\text{Concentration}\;\text{of}\;\text{Analyte}\left(\text{TCEQ}\;\text{or}\;\text{NNMA}\right)\text{in}\;\text{Aerosol}\;\left(\frac{\mu g}{\text{device}}\right)}{\text{Estimated Concentration of Analyte}\left(\text{TCEQ or NNMA}\right)\text{in JUULpod eLiquid }\left(\frac{\mu g}{\text{device}}\right)}\times 100\%$$ (2)

Because no reference standard was available for TCEQ or NNMA, e-liquid removed from 28 months aged unflavored JUULpods containing TCEQ and NNMA were used as the spiking solution to experimentally determine an approximate method LOQ (#5 in Figure 1). Fifty mg of the unflavored e-liquid containing TCEQ and NNMA was spiked into 1 mL of collected aerosol from both aged Virginia Tobacco 3.0% and Menthol 5.0% JUULpods. To investigate TCEQ and NNMA response over a range of concentrations in the aerosol matrix (i.e., flavors associated with Virginia Tobacco and Menthol), the spiked matrix was diluted 1:1, 1:4 and 1:40 using ultrapure water. Samples were analyzed according to the Agilent Technologies Ultivo Triple Quadrupole LC-MS/MS method as shown in Table 2.

## 3 Results

### 3.1 Confirmation analysis of TCEQ and NNMA in unflavored aged JUULpods

**FIGURE 3:**
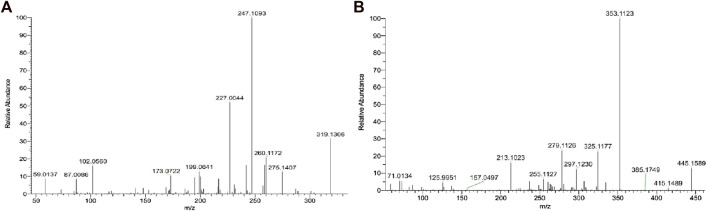
LC-HR-MS/MS Spectra for Compounds 1 [**(A)**; RT 1.86 min; TCEQ] and 2 [**(B)**; RT 2.49 min; NNMA].

For the analysis of unflavored e-liquid in aged JUULpods, the high resolving power of the orbitrap mass analyzer (240,000 m/Δm) was able to detect both leachable compounds, TCEQ and NNMA, at retention times of 1.86 min and 2.49 min, respectively. Figure 3 shows the mass spectra for each compound in which the high-resolution molecular mass (deprotonated molecular ions shown in Figure 3) and fragmentation patterns were consistent with the proposed structures and compound rationalizations for TCEQ and NNMA reported in the simulated leachable studies performed at WuXi AppTec., Inc. Efforts were made to elucidate the origin of these two compounds back to the source materials, however, they were not completely rationalized. Structural moieties for TCEQ were consistent with glycerin while structural moieties for NNMA were consistent with nicotine-related compounds.

**FIGURE 4:**
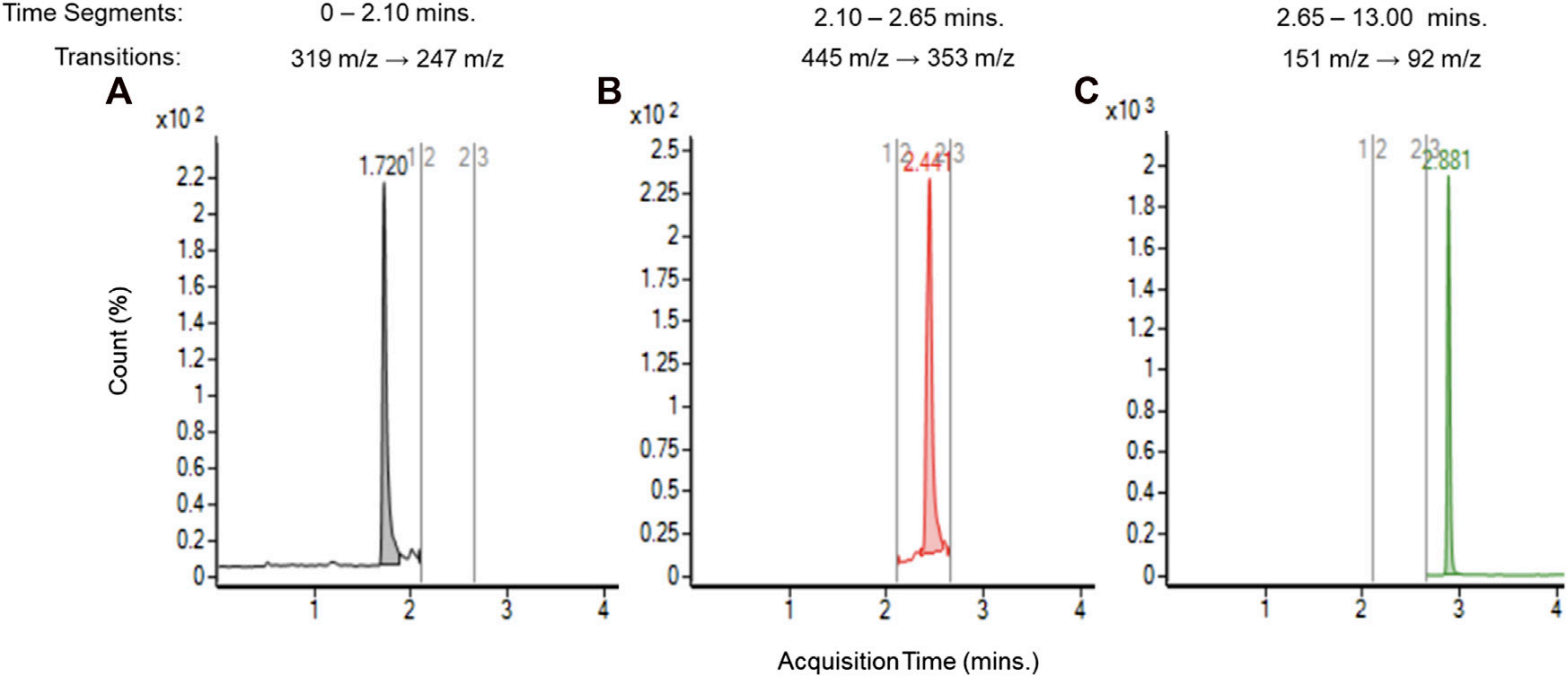
LC-MS/MS chromatograms for unflavored e-liquid from aged JUULpods stored at ambient conditions (25°C/60% RH) for 28 months and peaks associated with TCEQ [**(A)**, 1.7 min], NNMA [**(B)**, 2.4 min] and ISTD [**(C)**, 2.9 min].

After confirming the presences of TCEQ and NNMA, the method was transferred to an Agilent Technologies Ultivo triple quadrupole MS better suited for estimating concentration via internal standard. Multiple reaction monitoring (MRM) was performed using transitions from the deprotonated molecular ion to the base fragment for each compound via collision-induced dissociation (CID), 319 m/z to 247 m/z for TCEQ, 445 m/z to 353 m/z for NNMA and 151 m/z to 92 m/z for the internal standard (methylparaben at 40 ppb). Figure 4 depicts the chromatogram at each of the three discrete time-segmented MRM transitions for the unflavored base e-liquid formulation from aged JUULpods stored for 28 months at ambient conditions (25°C/60% RH) and the peaks associated with TCEQ (left, 1.7 min), NNMA (middle, 2.4 min) and ISTD (right, 2.9 min).

Using Eq. 1, concentrations of TCEQ and NNMA were estimated in the unflavored e-liquid removed from ambient aged JUULpods. Concentrations ranged from 0.118 to 0.123 µg/device for TCEQ and 0.105–0.151 µg/device for NNMA. The estimated concentrations determined in the LC-MS/MS analysis using the triple quadrupole method yielded results similar to those reported in the simulated leachable studies (see Table 1 estimated concentrations). The observed differences could be attributed to storage conditions (i.e., long term ambient vs. accelerated). The method specificity for e-liquid analysis provided confidence that the method was fit-for-purpose for the investigation and analysis of TCEQ and NNMA in aerosol collected from JUULpods.

### 3.2 Analysis of JUULpod aerosol for TCEQ and NNMA

**FIGURE 5:**
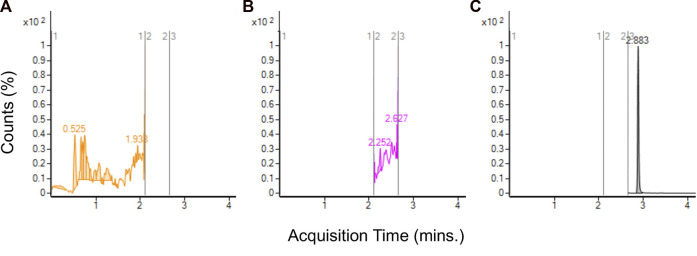
LC-MS/MS chromatograms for aerosol collected from unflavored base e-liquid formulation from aged JUULpods stored at ambient conditions (25°C/60% RH) for 28 months and no detectable peaks for TCEQ [**(A)**, absent at approximately 1.72 min] and NNMA [**(B)**, absent at approximately 2.44 min]. ISTD shown by **(C)** at 2.883 min].

Analysis of aerosol from JUULpods containing the ambient aged unflavored e-liquid found no detectable amounts of TCEQ or NNMA. Figure 5 shows the chromatograms at each time segment corresponding to TCEQ, NNMA and the ISTD in which no peak was observed for either leachable compound (refer to Figure 3 for peaks observed in e-liquid for comparison). In addition to unflavored JUULpods, aerosol was collected from 3 separate batches of commercially available Virginia Tobacco and Menthol JUULpods (both 5.0% and 3.0% nicotine-levels) after aging in ambient long-term storage for approximately 2.5–3 years (see Supplementary Table S1). No TCEQ or NNMA were detected in any of the aerosol samples analyzed in this study.

### 3.3 Determination of method limits and transfer efficiency of TCEQ and NNMA

**FIGURE 6:**
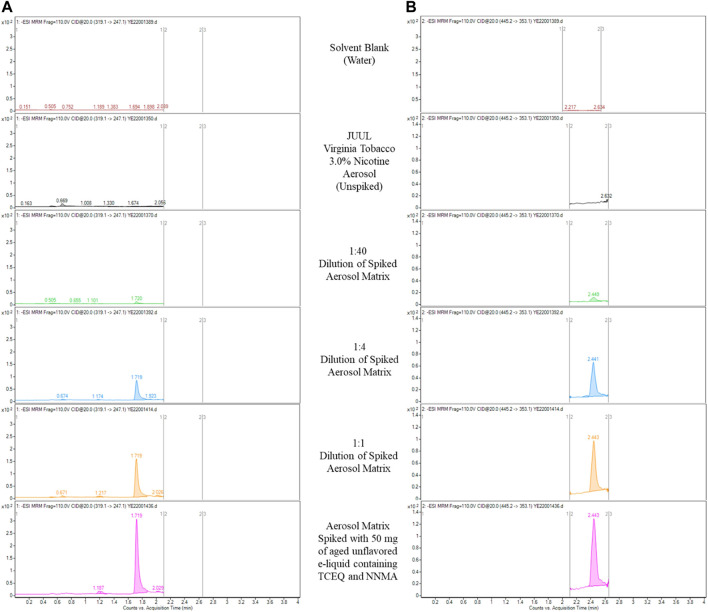
LC-MS/MS chromatograms for solvent blank and aerosol collected from commercial Virginia Tobacco (3% nicotine) JUULpods stored at ambient conditions (25°C/60% RH) for 3 years spiked with unflavored base e-liquid formulation containing TCEQ [**(A)**, 1.72 min] and NNMA [**(B)**, 2.44 min] and subsequent dilutions.

Although thorough evaluation of each chromatogram was appropriate from a qualitative analysis standpoint (detected vs. non-detected), comprehensive toxicological evaluations require semi-quantitative reported values to perform risk assessment. In cases where compounds are not detected, a conservative approach is to utilize the reported method limits (USEPA, 1991). However, no commercial reference standards for TCEQ or NNMA were available, nor any reasonable synthetic route possible due to the molecular size and structural complexity of the compounds. Therefore, an alternative analytical approach for the determination of method limits was used. Instead of using an authentic standard for spike recovery per ICH guidance (ICH, 2005), unflavored e-liquid removed from aged JUULpods, containing TCEQ and NNMA, was used as the spiking solution. Figure 6 shows the chromatograms of aerosol collected from Virginia Tobacco 3.0% nicotine JUULpods in which no detectable levels of TCEQ or NNMA were present. To investigate TCEQ and NNMA responses over a range of concentrations, serial dilutions of the spiked matrix was performed to determine the lowest analyte signal that provided reproducible results. A dilution of 1:40 spiked matrix, equivalent to 1.2 mg of aged unflavored e-liquid formulation in 1 mL of aerosol matrix, provided a peak area suitable for experimentally determining the LOQ per Eq. 1. The calculated experimental LOQs for TCEQ and NNMA were determined to be 0.003 µg/device for both compounds. Because all values for aerosol samples collected from aged JUULpods show no trace or detectable levels of TCEQ or NNMA, the experimentally determined LOQ of 0.003 µg/device was used for the calculation of the transfer efficiency according to Eq. 2. The transfer efficiency for TCEQ and NNMA based on estimated concentrations was calculated to be approximately 2.0%–2.8% according to Eq. 2. The observed transfer efficiency results for TCEQ and NNMA were expected based on the molecular mass and proposed structure of each non-volatile organic compound coupled with the operating temperatures associated with ENDS products (Alston, 2019).

## 4 Discussion

We describe a novel analytical approach to help estimate the user relevant exposure for data deficient leachables identified in simulated leachable studies of ENDS e-liquid. The approach is both practical and logical with the initial steps involving the identification, confirmation, and reproduction of two leachable compounds tentatively identified in aged unflavored e-liquid removed from JUULpods. Upon verification that the leachable compounds were present in aged e-liquid and analytical methods were fit-for-purpose, investigation of aerosol generated from JUULpods containing aged unflavored e-liquid was performed to better understand the transfer efficiency of these leachables, TCEQ and NNMA. Aerosol analysis indicated no detectable levels of either leachable compound was observed. In addition, aerosol generated from aged JUULpods (long term ambient storage for 3 years) containing Virginia Tobacco (5.0% and 3.0% nicotine) and Menthol (5.0% and 3.0% nicotine) e-liquid was also analyzed in which no detectable levels were observed. Finally, to provide semi-quantitative reported values, method limits of quantitation were established using an alternative approach to traditional ICH guidelines (ICH, 2005) due to no commercially available reference material or viable synthetic route. The novel analytical approach provided experimentally determined LOQs of 0.003 µg/device for each leachable compound in which estimated transfer efficiencies were calculated to be less than 3%. These experimentally determined LOQs provided semi-quantitative values to serve as exposure assumptions in the toxicological risk assessment of TCEQ and NNMA. While the focus in this case study was on two specific leachable compounds with respect to ENDS products, the approach can be applicable to many different non-targeted analytical analyses across a multitude of disciplines. To conclude, our aim in providing details discussed in the presented work is to help fill in the gaps in the existing non-targeted analysis of leachable compounds and toxicological risk assessment paradigm that remains in the ENDS industry.

## Data Availability

The datasets presented in this article are not readily available because the data supporting the conclusion of this article was used in support of regulatory filings and may be made available upon request and appropriate legal review. Requests to access the datasets should be directed to CS, cameron.smith@juul.com.
