# Corrigendum: Analytical approaches for the evaluation of data deficient simulated leachable compounds in ENDS products: a case study

**DOI:** 10.3389/fchem.2023.1334736

**Published:** 2023-11-28

**Authors:** Cameron Smith, Matthew Lyndon, Lena Jeong, Danielle Lehman, J. Brian Jameson, Harish Chevva, Felix Ayala-Fierro, David Cook, Karen Carter, Michael Oldham, I. Gene Gillman

**Affiliations:** Juul Labs Inc., Washington, DC, United States

**Keywords:** ENDS, e-cigarette, electronic cigarette, nicotine, leachables, risk assessment, aerosol

In the published article, there was an error in Eq. 1: **Calculation used for Analytical Reporting of TCEQ and NNMA** as published. A sample dilution factor was omitted, and therefore, values were overestimated by a factor of approximately 40. The equation previously stated:

$$\begin{array}{c} \frac{\text{Analyte}\;\left(\text{TCEQ}\;\text{or}\;\text{NNMA}\right)\;\text{Peak}\;\text{Area}}{\text{ISTD}\;\text{Peak}\;\text{Area}}\times \text{ISTD Conc}.\left(0.04\frac{\mu g}{mL}\right)\times \text{Fill Wt}.\text{in JUULpod}\left(780\frac{mg}{Device}\right)\\ x{\text{Density}}^{-1}\left(0.8430\frac{mL}{mg}\right)=Estimated\;Concentration\;of\;TCEQ\;or\;NNMA\;\left(\frac{\mu g}{\text{Device}}\right) \end{array}$$ (1)

The corrected equation is shown below. The e-liquid weight (approximately 50 mg or 0.05 g) in 1 mL ultrapure water containing internal standard was used in the updated equation (per **2 Experimental methods**, *2.3 e-liquid sample preparation and analysis using LC-HR-MS/MS and LC-MS/MS negative mode ESI* in the original publication), and density was removed as the weight to volume is addressed. Note, fill wt. in JUULpod was modified from “780 mg” to “0.780 g” to keep units consistent; however, these are the same values.

$$\begin{array}{c} \frac{\text{Analyte}\;\left(\text{TCEQ}\;\text{or}\;\text{NNMA}\right)\;\text{Peak}\;\text{Area}}{\text{ISTD}\;\text{Peak}\;\text{Area}}\times \text{ISTD Conc}.\left(0.04\frac{\mu g}{mL}\right)\times \frac{\text{Sample}\;\text{Volume}\;\left(\text{mL}\right)}{e-Liquid\;Aliquit\;Wt.\left(g\right)}\times \\ \text{Fill Wt}.\text{in JUULpod}\left(0.780\frac{gram}{Device}\right)=Estimated\;Concentration\;of\;TCEQ\;or\;NNMA\;\left(\frac{\mu g}{\text{Device}}\right) \end{array}$$ (1)

Regardless of the estimated concentrations in the e-liquid, the analytical approach for the calculation of the transfer efficiency discussed in the publication (**3 Results**, *3.3 Determination of method limits and transfer efficiency of TCEQ and NNMA*) is based on a 1:40 dilution. A hypothetical example below is used to illustrate the point using **Eq. 2**: **Calculation of Transfer Efficiency for TCEQ and NNMA**.

$$\frac{\text{Esitmated}\;\text{Concentration}\;\text{of}\;\text{Analyte}\left(\text{TCEQ}\;\text{or}\;\text{NNMA}\right)\text{in}\;\text{Aerosol}\;\left(\frac{\mu g}{\text{device}}\right)}{\text{Estimated Concentration of Analyte}\left(\text{TCEQ or NNMA}\right)\text{in JUULpod eLiquid }\left(\frac{\mu g}{\text{device}}\right)}\times 100\%$$

Example:

$$\frac{0.25\text{or}0.025\text{or}0.0025\left(\frac{\mu g}{\text{device}}\right)}{10\text{or}1.0\text{or}0.1\left(\frac{\mu g}{\text{device}}\right)}\times 100\%=2.5\%$$

If estimated concentrations in the e-liquid were 10, 1.0 or 0.1 and diluted by 40 to achieve an experimental LOQ of 0.25, 0.025 or 0.0025 µg/device, respectively, the resulting transfer efficiency remains 2.5%. As stated in the publication, the analytical approach is useful when traditional analytical approaches that utilize reference standards are not available and semi-quantitative values are needed for risk assessment.

The updated equation was used to update several values reported in the original publication (see updated text corrections below). Note, no additional data was collected, nor any additional experiments performed.

A correction has been made to section **3 Results**, *3.1 Confirmation analysis of TCEQ and NNMA in unflavored aged JUULpods*, 3rd paragraph. The affected sentences previously stated:

“Using Eq. 1, concentrations of TCEQ and NNMA were estimated in the unflavored e-liquid removed from ambient aged JUULpods. Concentrations ranged from 4.7 to 5.7 µg/device for TCEQ and 4.9–6.0 µg/device for NNMA.”

The corrected sentence appears below:

“Using Eq. 1, concentrations of TCEQ and NNMA were estimated in the unflavored e-liquid removed from ambient aged JUULpods. Concentrations ranged from 0.118 to 0.123 µg/device for TCEQ and 0.105–0.151 µg/device for NNMA.”

A correction has been made to section **3 Results**, *3.3 Determination of method limits and transfer efficiency of TCEQ and NNMA*. The affected sentences previously stated:

“The calculated experimental LOQs for TCEQ and NNMA were determined to be 0.12 µg/device for both compounds. Because all values for aerosol samples collected from aged JUULpods showed no trace or detectable levels of TCEQ or NNMA, the experimentally determined LOQ of 0.12 µg/device was used for the calculation of the transfer efficiency according to **Eq. 2**. The transfer efficiency for TCEQ and NNMA based on estimated concentrations from the simulated leachable study (see Table 1) were calculated to be approximately 1.6% according to **Eq. 2**.”

The corrected sentences appears below:

“The calculated experimental LOQs for TCEQ and NNMA were determined to be 0.003 µg/device for both compounds. Because all values for aerosol samples collected from aged JUULpods show no trace or detectable levels of TCEQ or NNMA, the experimentally determined LOQ of 0.003 µg/device was used for the calculation of the transfer efficiency according to **Eq. 2**. The transfer efficiency for TCEQ and NNMA based on estimated concentrations was calculated to be approximately 2.0%–2.8% according to **Eq. 2**.”

A correction has been made to section **4 Discussion**. The sentence previously stated:

“The novel analytical approach provided experimentally determined LOQs of 0.12 µg/device for each leachable compound in which estimated transfer efficiencies were calculated to be less than 2%.”

The corrected sentence appears below:

“The novel analytical approach provided experimentally determined LOQs of 0.003 µg/device for each leachable compound in which estimated transfer efficiencies were calculated to be less than 3%.”

A correction has been made to the **Abstract**. The sentence previously stated:

“The transfer efficiency of each leachable compound was experimentally determined to be less than 2% based on the limit of quantitation, which then could be used to define a relevant exposure limit for the toxicological risk assessment.”

The corrected sentence appears below:

“The transfer efficiency of each leachable compound was experimentally determined to be less than 3% based on the limit of quantitation, which then could be used to define a relevant exposure limit for the toxicological risk assessment.”

In the published article, there was an error in Table 1 as published. The original table read, “Estimated Concentration at 30°C/65% RH for 22 weeks (Simulated 18-month aging)” (1st column, 5th row).

The corrected Table 1 appears below.

**TABLE 1:** Information on data deficient leachable compounds.

Name	Compound 1 [RT 1.71 min]	Compound 2 [RT 2.44 min]
CAS #	Not Given	Not Given
Molecular Formula	C_16_H_20_N_2_O_5_	C_18_H_24_N_2_O_4_
Molecular Weight	320.1360	446.1686
Estimated Concentration at 30°C/65%RH for 22 weeks (Simulated 9-month aging)	1.1 ± 0.1 µg/device	2.0 ± 0.1 µg/device
Estimated Concentration at 40°C/75% RH for 22 weeks (Simulated 18-month aging)	8.5 ± 0.7 µg/device	6.2 ± 0.4 µg/device
Structural Characteristics	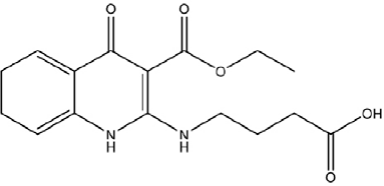	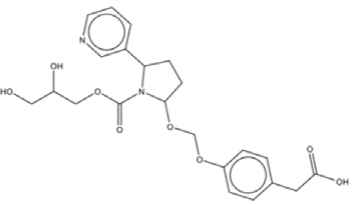
Reported Tentative Compound Identification	1,8,9-Trihydro-2-(3-carboxypropylamine-N-yl)-3-ethylcarboxylate-4-quinolone	Nornicotine, N-carboxyglycerol-5′- [methoxy-1-(ρ-hydroxybenzene-*O*4-yl-acetic acid)]
Abbreviation for Narrative	TCEQ	NNMA

The authors apologize for these errors and state that this does not change the scientific conclusions of the article in any way. The original article has been updated.

