# Supplementary material for: Analytical approaches for the evaluation of data deficient simulated leachable compounds in ENDS products: a case study
Source: Front Chem. 2023 Aug 4;11:1212744. doi: 10.3389/fchem.2023.1212744 (PMC10436322; doi:10.3389/fchem.2023.1212744)
Supplement: Supplementary file 1 [file DataSheet1.docx]

Supplementary Material

Analytical Approaches for the Evaluation of Data Deficient Simulated Leachable Compounds in ENDS Products: A Case Study

Cameron Smith^1*^, Matthew Lyndon^1^, Lena Jeong^1^, Danielle Lehman^1^, J. Brian Jameson^1^, Harish Chevva^1^, Felix Ayala-Fierro^1^, David Cook^1^, Karen Carter^1^, Michael Oldham^1^, I. Gene Gillman^1^

^1^JUUL Labs, 1000 F Street NW, Washington, DC 20004

*** Correspondence:** Cameron Smith: cameron.smith@juul.com

# Supplementary Method

*Simulated Leachable Studies (WuXi App Tec., Inc.)*

For GC-MS analysis, unflavored e-liquid removed from aged JUULpods was performed for GC-MS analysis of volatiles and semi-volatiles. The column used was Agilent DB-1MS UI (30 m x 0.25 mm x 0.25 µm with 1.0 mL min^-1^ constant helium flow. Initial oven temperature was set to 40°C with a 1 min. hold time and a 10°C min^-1^ ramp to a final temperature of 300°C with a 10 min. hold time. The inlet temperature was 210°C in pulsed splitless mode and a 1.0 µL injection volume. Detector settings for electron impact (EI) mode were generic (transfer line temp. 280°C, source temp. 230°C, quad. temp. 150°C) with a mass range covering 35 to 650 m/z and a 4.5 second solvent delay. An instrument autotune was completed successfully prior to analysis as well as system suitability assessment using known reference standards to ensure the instrument was performing properly throughout the analysis. The system was found to be performing properly by the positive identification of each of the standards using the NIST library and retention time.

Compounds were reported using the following basic criteria: four main ingredients in the e-liquid were excluded (PG, VG, nicotine, and benzoic acid); Compounds were detected and had similar retention times in all replicates; Compounds were semi-quantitated when ≥0.75 µg device^-1^. The compounds were determined using the NIST library within the MassHunter software as well as scientific judgment. Score was used to determine the match quality and was a determination of how close the unknown spectrum matches the library spectrum. The score was calculated based on the database match using fragment and parent ions and their relative abundances. The scale was 0-100, with 100 being the best possible match. When a library match could not be obtained, manual identification was performed using the mass data and a score was not applicable. Due to the fact that similar compounds display similar fragmentation, individual alkanes, alkenes, alcohols and other like-class compounds may not be distinguishable from other members of their respective class. The compounds listed are identified by the library match but may represent another compound in the same class. Some compounds may be reported by class only due to the interpretation of fragmentation by the software algorithm.

All compounds identified were semi-quantitated against the reference standard using the following equation:

**Equation S1.**


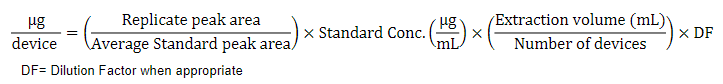


For LC-MS analyses, direct injection of unflavored e-liquid removed from aged JUULpods was performed on Agilent 1290 UHPLC coupled with an Agilent Technologies 6530 Q-TOF for the analysis of semi-volatiles and non-volatiles in both positive and negative ESI mode. Chromatographic separations were performed on an Agilent Zorbax RRHD SB-C18, 1.8 µm, 2.1 x 100 mm heated to 40°C, 5 µL injection volume and 0.5 mL min-1. flow rate. For positive ESI mode, mobile phases were 0.1% formic acid in water (A) and 0.1% formic acid in methanol (B). For negative ESI mode, mobile phases were 0.1% acetic acid in water (A) and 0.1% acetic acid in methanol (B). The gradient program was as follows: 5% B (0-0.75 mins.), 5-95% B (0.75-3 mins.), 95% B (3-19 mins.), 95-100% B (19-19.1 mins), 100% B (19.1-28 mins.), 100-5% B (28-28.2 mins.), 5% B (28.2-30 mins.). The LC-MS was autotuned and calibrated prior to analysis. A reference mass solution was infused into the source to ensure mass accuracy throughout the analysis. System suitability was assessed using a prepared standard mix (12-aminododecanolactam, di-n-octyl phthalate, irganox 1010, irganox 1076, methylparaben, and trioctyl trimellitate) to ensure the instrument and semi-quantitation was performing properly.

Compounds were reported using the following basic criteria: four main ingredients in the e-liquid were excluded (PG, VG, nicotine, and benzoic acid); Compounds were detected and had similar retention times in all replicates; Compounds were semi-quantitated when ≥0.75 µg device-1. Data extraction was performed by extracting mass spectra for each peak. The positive and negative ion features were searched against the WuXi AppTec., Inc. E&L database and mass spectral library. Additional tools were used in the identification of compounds, including, but not limited to literature searches and structural elucidation via extraction and interpretation of fragmentation data. All compounds identified by mass spectral library search, database search, and/or molecular formula generator (MFG) were semi-quantitated against di-n-octyl phthalate (positive ion mode) or methylparaben (negative ion mode), the preparation parameters, and any applicable dilution. Semi-quantitation was performed using the equation 1 as shown above.

# Supplementary Table(s)

**Table S1.** Sample Information for the Analysis of TCEQ and NNMA in JUULpod aerosol.

| Samples | Pod Fill Date  (MM/DD/YYYY) | Storage Condition | Product Age Prior to Testing |
| --- | --- | --- | --- |
| Unflavored e-Liquid*  (PG/VG/Nicotine/Benzoic Acid) | 02/21/2020 | Storage Facility  Precision Stability Storage  (Wilson, NC)    Temperature: 25°C  Relative Humidity: 60% | 2Y 4M 8D |
| Virginia Tobacco 5% - Batch 1 | 06/13/2019 |  | 3Y 0M 16 D |
| Virginia Tobacco 5% - Batch 2 | 06/13/2019 |  | 3Y 0M 16 D |
| Virginia Tobacco 5% - Batch 3 | 06/14/2019 |  | 3Y 0M 15 D |
| Virginia Tobacco 3% - Batch 1 | 06/24/2019 |  | 3Y 0M 5 D |
| Virginia Tobacco 3% - Batch 2 | 06/24/2019 |  | 3Y 0M 5 D |
| Virginia Tobacco 3% - Batch 3 | 06/24/2019 |  | 3Y 0M 5 D |
| Menthol 5% - Batch 1 | 07/12/2019 |  | 2Y 11M 17D |
| Menthol 5% - Batch 2 | 07/12/2019 |  | 2Y 11M 17D |
| Menthol 5% - Batch 3 | 07/12/2019 |  | 2Y 11M 17D |
| Menthol 3% - Batch 1 | 08/20/2019 |  | 2Y 10M 9D |
| Menthol 3% - Batch 2 | 08/20/2019 |  | 2Y 10M 9D |
| Menthol 3% - Batch 3 | 08/20/2019 |  | 2Y 10M 9D |

*Only sample analyzed for TCEQ and NNMA in e-liquid removed from aged JUULpod.
